# Genomic characterization and comparative genomic analysis of HS-associated *Pasteurella multocida* serotype B:2 strains from Pakistan

**DOI:** 10.1186/s12864-023-09626-5

**Published:** 2023-09-14

**Authors:** Sadia Mahboob, Nimat Ullah, Muhammad Farhan Ul Haque, Waqar Rauf, Mazhar Iqbal, Amjad Ali, Moazur Rahman

**Affiliations:** 1 Health Biotechnology Division, National Institute for Biotechnology and Genetic Engineering College, Pakistan Institute of Engineering and Applied Sciences (NIBGE-C, PIEAS), Faisalabad, 38000 Punjab Pakistan; 2 Atta-Ur-Rahman School of Applied Biosciences (ASAB), National University of Sciences and Technology (NUST), Islamabad, 44000 Pakistan; 3 School of Biological Sciences, University of the Punjab, Lahore, 54590 Pakistan; 4 Centre of Excellence in Molecular Biology, University of the Punjab, Lahore, 53700 Pakistan

**Keywords:** Haemorrhagic septicaemia, *Pasteurella multocida*, Virulence gene profiling, *PlpE*, Serotype B:2

## Abstract

**Background:**

Haemorrhagic septicaemia (HS) is a highly fatal and predominant disease in livestock, particularly cattle and buffalo in the tropical regions of the world. *Pasteurella multocida* (*P. multocida*), serotypes B:2 and E:2, are reported to be the main causes of HS wherein serotype B:2 is more common in Asian countries including Pakistan and costs heavy financial losses every year. As yet, very little molecular and genomic information related to the HS-associated serotypes of *P. multocida* isolated from Pakistan is available. Therefore, this study aimed to explore the characteristics of novel bovine isolates of *P. multocida* serotype B:2 at the genomic level and perform comparative genomic analysis of various *P. multocida* strains from Pakistan to better understand the genetic basis of pathogenesis and virulence.

**Results:**

To understand the genomic variability and pathogenomics, we characterized three HS-associated *P. multocida* serotype B:2 strains isolated from the Faisalabad (PM1), Peshawar (PM2) and Okara (PM3) districts of Punjab, Pakistan. Together with the other nine publicly available Pakistani-origin *P. multocida* strains and a reference strain Pm70, a comparative genomic analysis was performed. The sequenced strains were characterized as serotype B and belong to ST-122. The strains contain no plasmids; however, each strain contains at least two complete prophages. The pan-genome analysis revealed a higher number of core genes indicating a close resemblance to the studied genomes and very few genes (1%) of the core genome serve as a part of virulence, disease, and defense mechanisms. We further identified that studied *P. multocida* B:2 strains harbor common antibiotic resistance genes, specifically *PBP3* and *EF-Tu*. Remarkably, the distribution of virulence factors revealed that *OmpH* and *plpE* were not present in any *P. multocida* B:2 strains while the presence of these antigens was reported uniformly in all serotypes of *P*. *multocida.*

**Conclusion:**

This study's findings indicate the absence of *OmpH* and *PlpE* in the analyzed *P. multocida* B:2 strains, which are known surface antigens and provide protective immunity against *P. multocida* infection. The availability of additional genomic data on *P. multocida* B:2 strains from Pakistan will facilitate the development of localized therapeutic agents and rapid diagnostic tools specifically targeting HS-associated *P. multocida* B:2 strains.

**Supplementary Information:**

The online version contains supplementary material available at 10.1186/s12864-023-09626-5.

## Background

*Pasteurella multocida* (*P. multocida*) is a Gram-negative, facultative anaerobe and an economically important veterinary pathogen. It exists both as a commensal and an opportunist pathogen, found in nasopharyngeal microflora as well as in the proximal gastrointestinal tract of animals [1]. It is responsible for a variety of acute to chronic respiratory infections in diverse wild and domestic animal species including poultry, livestock and pet animal throughout the world [2–4]. It was the first species of the genus *Pasteurella* isolated by Louis Pasteur in 1880 and was found to cause fowl cholera in birds [5]. Over a period of more than a century, it is well established that *P. multocida* plays a principal role in inducing serious infections like haemorrhagic septicaemia (HS) in cattle and buffaloes [6, 7], bovine respiratory disease (BRD) in calves [8], fowl cholera (FC) in poultry and porcine atrophic rhinitis (AR) in pigs and rabbits [9].

HS is an acute and highly fatal disease in cattle and water buffaloes [2]. It is a specific form of septicemic pasteurellosis mainly caused by *P. multocida* serotypes B:2 (predominant in Asia) and E:2 (predominant in Africa). During the period between 2005–2019, varying rates of HS infection were documented in cattle across different parts of the world: 100% in America and Europe, 85.4% in Africa, and 67.2% in Asia [10]. The highest disease incidence has been reported in South and Southeast Asian countries including Bhutan, China, India, Indonesia, Mongolia, Myanmar, Philippines, Sri Lanka and Malaysia [11–13]. Outbreaks of HS are common in Cambodia with high mortality rates in buffaloes (98%) and cattle (97%) [14]. Similarly, sporadic epidemics in South Asia countries mostly occur throughout in the year affecting large herd populations [15–17]. It has been considered an economically significant disease because of the higher mortality rate (80–100%) in infected animals as compared to other infectious diseases [7]. The seroprevalence of HS has been extensively reported in Pakistan [16, 18, 19]. In this context, buffaloes exhibit greater average morbidity (12.56%) and mortality (22.44%) compared to cattle (with respective rates of 2.42% and 6.46%) [20], indicating that buffaloes are more susceptible to HS [21]. The annual economic losses due to HS have been estimated at around $350 million in Pakistan in 2002 [13].

Antibiotic treatments have been found effective only if the treatment started soon after the diagnosis of HS infection at early stages [22]. Antimicrobials including oxytetracycline, sulfamethoxazole, a combination of streptomycin and penicillin, and sulphaquinoxaline are being used to treat HS [12]. However, these treatments are associated with a significant cost. Moreover, HS progresses rapidly, and therefore, antibiotic therapy is often unsuccessful at a later stage [23]. On the other hand, unnecessary overuse of antimicrobials has induced multi-drug resistance in the pathogens. Recent studies reported that strains of *P. multocida* are resistant to multiple antimicrobials commonly used to treat HS. These include amoxicillin, tetracycline, lincomycin, penicillin, oxytetracycline, chloramphenicol, gentamicin, and enrofloxacin [24, 25]. In a recent comparative genomic analysis of HS-associated strains from different geographical regions, including 12 from Pakistan, 2 from Thailand, and HS-associated North American strain M1404, alongside non-HS strains (Pm70, 37,950, HN06, and 3480) identified 35 unique genes in Pakistani isolates TX1 and BUKK [26]. These unique genes encode elements having significant similarity with an integrative conjugative element (ICEPmu1), previously reported in strain 36,950 [27]. This element encodes several acquired antimicrobial resistance genes including aminoglycoside resistance genes *aph(3’)-lc*, *strA,* and *strB*, sulphonamide resistance gene *sul2*, beta-lactamase resistance gene *bla*_TEM-1B_, tetracycline resistance gene *tet(H)*, and *catA2* conferring resistance to chloramphenicol [26]. Though several antimicrobials are available, most of them are becoming ineffective against emerging multi-drug resistant *P. multocida* and posing challenges for treating HS infections.

Several genes encoding putative virulence factors (VFs) have been identified to date [4], with multiple studies indicating a correlation between the prevalence of VFs and the host-specific disease manifestation [28]. Molecular level studies reported the presence of *hsf1*, *hgbB*, *pfhA*, and *tbpA* while absence of *pmHAS*, *plpE*, and *tadD* in all bovine isolates [29]. In contrarily, *nanB, sodC, hgbA, exbB, ompH, ptfA, nanB and hbgA* were found present in B:2 strain of *P. multocida* causing fowl cholera in layer chicken in Bangladesh [30].

Unfortunately, there is currently very little molecular and genomic information available regarding the HS-associated serotypes of *P. multocida* strains isolated from Pakistan. Therefore, this study was aimed to explore the genomic characteristics of bovine isolates of *P. multocida* serotype B:2 from Pakistan. This involve conducting a comparative genomic analysis of various *P. multocida* strains from Pakistan and a reference strain Pm70. This analysis aimed to enhance our understanding of the genetic basis of pathogenesis, genetic diversity, virulence, and identification of cross-protective vaccine candidates for the prevailing strains.

## Results

### Characteristics of *P. multocida* isolates

**Fig. 1:**
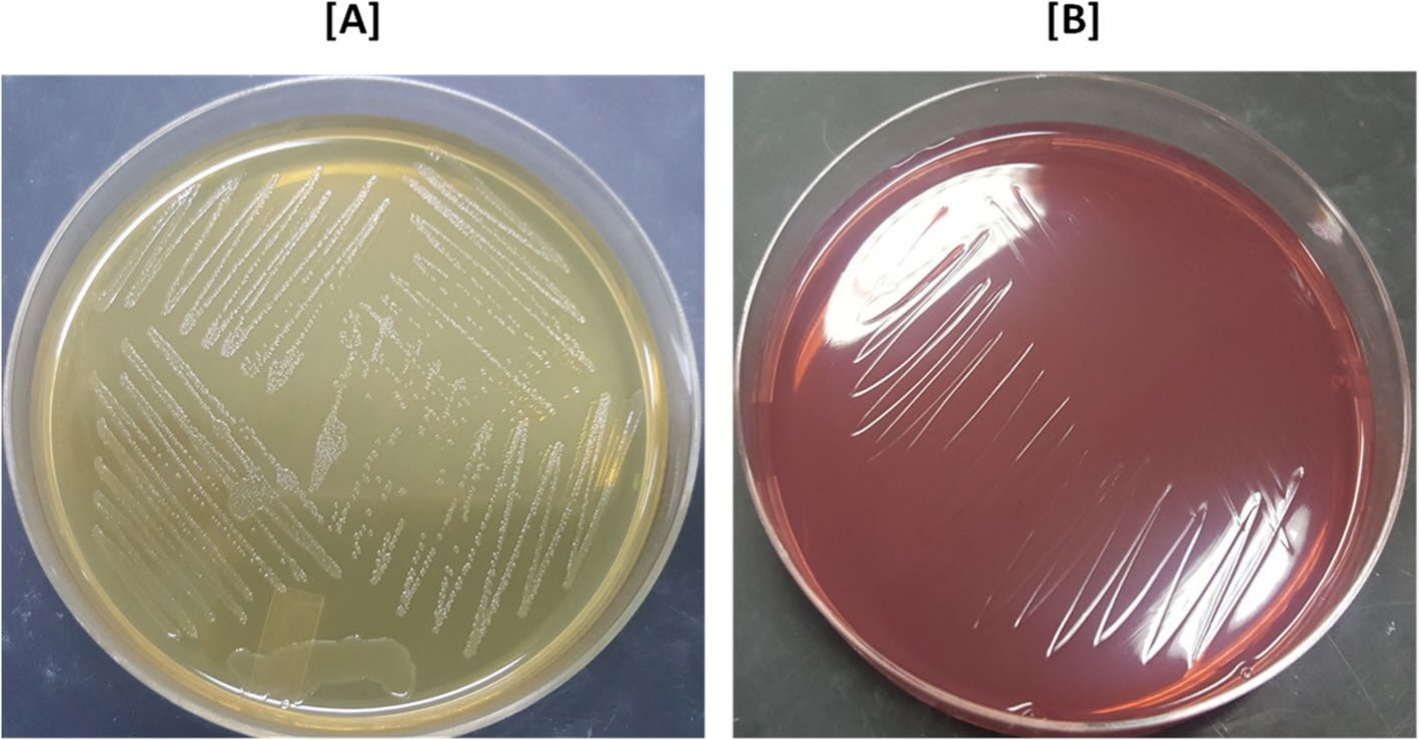
Morphological characteristics of *P. multocida* isolates on BHI and MacConkey agar plates. **A **Translucent and greyish colonies on BHI agar after 24 h, **B ** MacConkey agar plate showing no growth after 24 h

After 24 h of incubation at 37 ℃ very regular, transparent, small (approximately 1–1.5 mm in diameter) and greyish colonies were observed on brain heart infusion (BHI) agar plates while no growth was observed on MacConkey agar (Fig. 1).

**Fig. 2:**
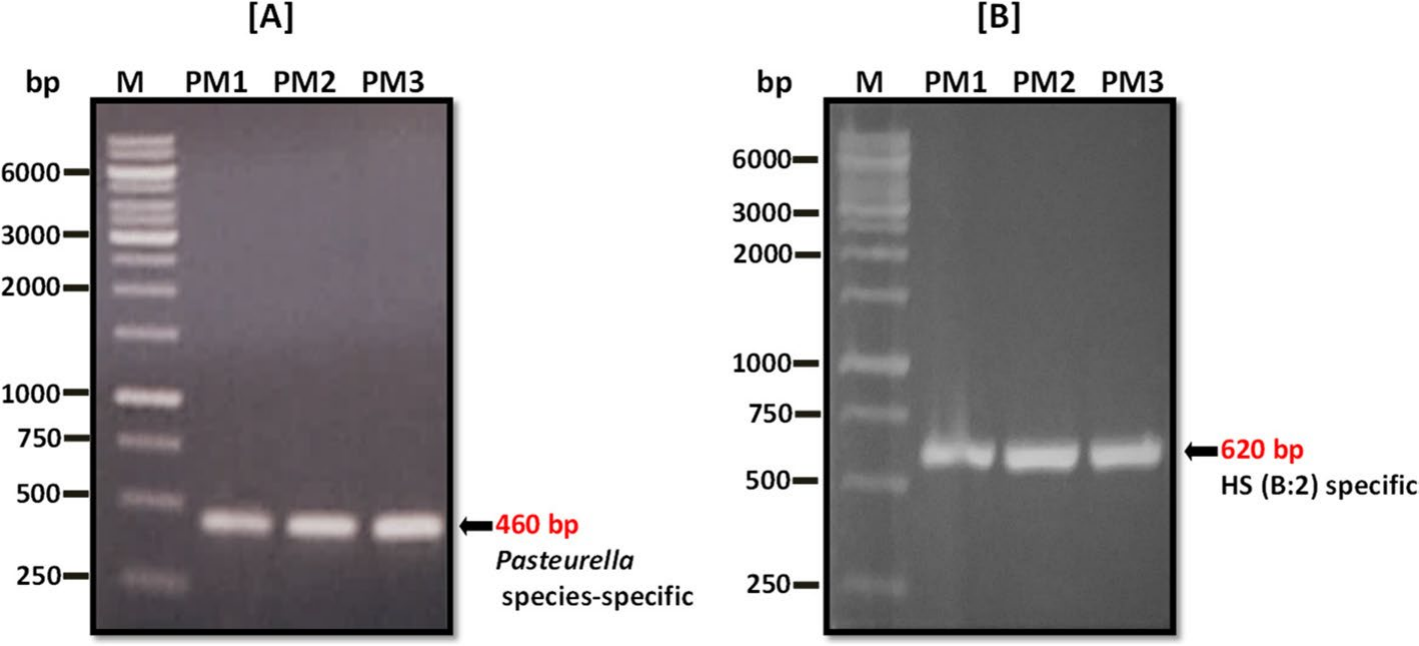
Molecular characterization of *P. multocida* isolates (PM1, PM2 and PM3). **A** The amplified product of *kmt*1 gene using *Pasteurella* species-specific primers and (**B**) amplified product of 6B gene using HS causing *P. multocida* B:2–type-specific primers. Full-length gels are presented in the Supplementary Figure (S1)

The *kmt*1 and 6B genes are unique DNA fragments that are used to distinguish *P. multocida* species and the serotype B:2. The *kmt*1 gene identifies *P. multocida* species (species-specific) whereas the 6B gene distinguishes HS causing B:2 serotype of *P. multocida* (type-specific). The PCR amplified *kmt*1 gene fragment (KMT1SP6-KMT1T7 primers) of size 460 bp revealed that all three isolates belong to *Pasteurella* species, while the amplification of 6B gene (KTSP61-KTT72 primers) specifically produced a fragment of 620 bp, confirmed that they belong to serotype B:**2 **(Fig. 2).

### Genomic features 

The genome sequencing of PM1 produced 806,011 reads, with a total read size of 490,054,688 bp, providing a 203-fold coverage of the entire genome. Likewise, for PM2, a total of 806,478 reads were generated, with a total read size of 409,690,824 bp, achieving high 178-fold coverage. Similarly, for PM3, a total of 6,986,762 reads were generated, with a total read size of 705,035,298 bp, attaining 299-fold coverage. The de novo assembly of PM1, PM2, and PM3 generated 128, 33, and 23 contigs with N50 values of 289,611 bp, 166,244 bp, and 289,471 bp, respectively and L50 values 3, 4, and 3, respectively. (Table 1). The cumulative genome size of PM1 is 2,413,477 bp, PM2 is 2,300,632 bp, and PM3 is 2,350,552 bp having 41%, 40.4%, and 40.3% G + C contents, respectively. Genome annotation predicted 2,377 genes in PM1, 2,192 in PM2, and 2,249 in PM3. These predicted genes were further characterized as 2,281 protein coding sequences (CDS) in PM1, 2,098 in PM2, and 2,158 in PM3. PM1 and PM2 harbor 8 rRNAs while PM3 contains 4 rRNAs. Four ncRNAs are present in each genome (Table 1).

**Table 1:** Genomic features of *P. multocida* isolates sequenced in this study

Features	PM1	PM2	PM3
Number of Reads	806,011	806,478	6,986,762
Total read size (bp)	490,054,688	409,690,824	705,035,298
Coverage	203	178	299
Total length (bp)	2,413,477	2,300,632	2,350,552
Contigs	128	33	23
N50	289,611	166,244	289,471
L50	3	4	3
% GC content	41	40.4	40.3
Genes (total)	2,377	2,192	2,249
CDS (with protein)	2,281	2,098	2,158
rRNA	4, 3, 1 (5S, 16S, 23S)	4, 3, 1 (5S, 16S, 23S)	2, 1, 1 (5S, 16S, 23S)
tRNA	52	51	47
ncRNA	4	4	4
Sequence type	Multi-host (ST-44), RIRDC (ST-122)	Multi-host (ST-44), RIRDC (ST-122)	Multi-host (ST-44), RIRDC (ST-122)
Accession number	JAEMBU000000000	JAFFJB000000000	SDAS00000000

The multi-host multi-locus sequence typing (MLST) scheme analyses assigned ST-44 to all sequenced strains as 100% nucleotide identity over alleles was observed for the genes *adk*, *aroA*, *deoD*, *gdhA*, *g6pd*, *mdh*, and *pgi*. On the other hand, the RIRDC MLST scheme showed that all the strains belong to ST-122 due to 100% nucleotide identity over alleles for the genes *adk*, *est*, *pmi*, *zwf*, *mdh*, *gdh*, and *pgi*.

### Identification of prophage regions and plasmids in the sequenced strains

The *P. multocida* strain PM1 contains 3 complete phage regions with lengths of 39.1Kb (Mannhe_vB_MhS_587AP2), 37.7Kb (Escher_D108), and 15.6Kb (Salmon_118970_sal3) (Table 2). Two complete prophages, Entero_Mu (37.7 Kb) and Salmon_118970_sal3 (30.4 Kb), along with one incomplete prophage, Pseudo_phiR18 (14.3 Kb), were found in strain PM2 (Table 2). Similarly, strain PM3 harbors two complete prophages, Escher_D108 (37.7 Kb) and Mannhe_vB_MhS_535AP2 (39.1 Kb), along with one incomplete prophage, Pseudo_phiR18 (14.3 Kb) (Table 2). No plasmid was identified in any sequenced genomes by the PlasmidFinder.

**Table 2:** Summary of prophage regions in the sequenced *P. multocida* strains

Strain	Prophage	Region Position	Completeness	Length	Number of total proteins	GC %	Accession No
**PM1**	Mannhe_vB_MhS_587AP2	492,476–531,610	Intact	39.1Kb	44	37.98	NC_028743
	Escher_D108	819,200–856978	Intact	37.7Kb	52	42.30	NC_013594
	Salmon_118970_sal3	2,013,721–2029419	Intact	30.4Kb	26	37.73	NC_031940
**PM2**	Entero_Mu	675,857–713,635	Intact	37.7 Kb	52	42.3	NC_000929
	Salmon_118970_sal3	2,127,539–2,157,945	Intact	30.4 Kb	26	37.73	NC_031940
	Pseudo_phiR18	2,189,398–2,203,706	Incomplete	14.3 Kb	20	42.09	NC_041964
**PM3**	Escher_D108	624,695–662,473	Intact	37.7Kb	52	42.30	NC_013594
	Mannhe_vB_MhS_535AP2	492,442–531,576	Intact	39.1 Kb	44	37.98	NC_028853
	Pseudo_phiR18	2,034,815–2049138	Incomplete	14.3 Kb	20	42.09	NC_041964

### Pan-genome estimation and phylogenetic analysis

**Fig. 3:**
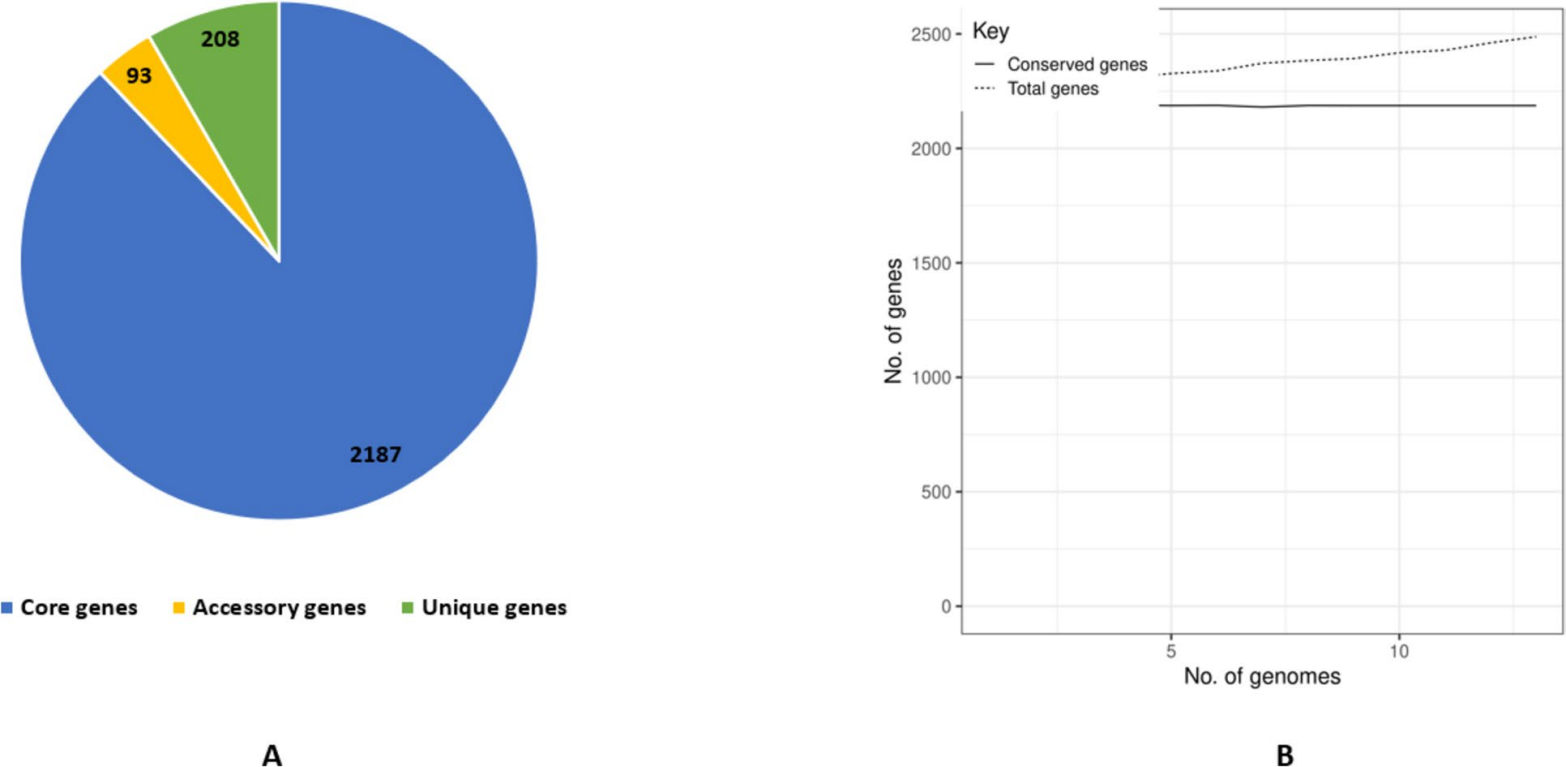
Pan-genome analysis of 12 Pakistani *P. multocida* strains (PM1, PM2, PM3, PVAcc, V1, TX1, Islm, Karachi, BUKK, Faisal, ATTK, and Pesh) and a reference strain Pm70. **A** Chart showing the number of core, accessory, and unique genes, **B** Plot of pan and core-genome

The pan-genome consists of 2,488 genes, of which 2187 are core, 93 accessory, and 208 unique genes (Fig. 3A). In pan-genome plot, the number of pan-genes increases with the increase of new genomes suggesting that the pan-genome of Pakistani *P. multocida* strains is relatively stable (Fig. 3B). In contrast, the core-genome curve becomes steady after adding certain genomes, indicating a stable core-genome (Fig. 3B).

**Fig. 4:**
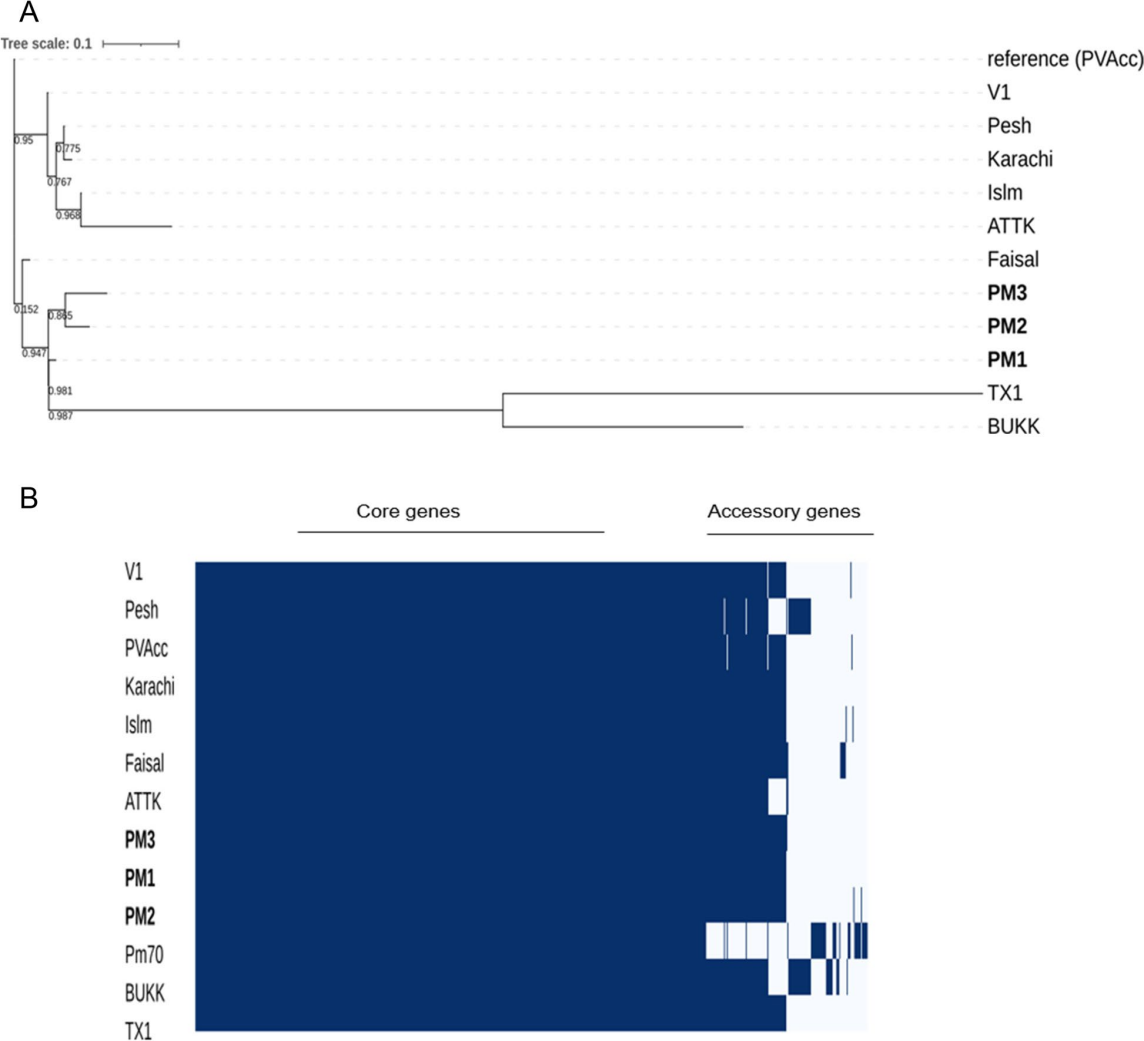
Whole genome SNPs-based phylogenetic relationship (**A**) and distribution of core and accessory genes (**B**) of the sequenced strains (PM1, PM2, and PM3) with 9 other Pakistani *P. multocida* strains (PVAcc, V1, TX1, Islm, Karachi, BUKK, Faisal, ATTK, and Pesh). The heatmap shows the presence and absence of genes

Phylogenetic analysis of PM1, PM2, and PM3 with other publicly available Pakistani *P. multocida* strains was performed based on whole-genome SNPs as well as core-genome SNPs. The whole-genome SNPs-based phylogenetic analysis revealed that the three newly sequenced strains, PM1, PM2, and PM3, form cluster together nearest to the previously sequenced strain Faisal **(**Fig. 4**)**. Additionally, a core-genome SNP-based phylogenetic analysis was also performed (Supplementary Figure S2). Following an extensive comparison of the phylogenetic trees, it was observed that the whole-genome SNP-based phylogeny demonstrated consistency with the core-genome SNP-based phylogeny and provided comparable results.

### Functional annotation of core and unique genes

**Fig. 5:**
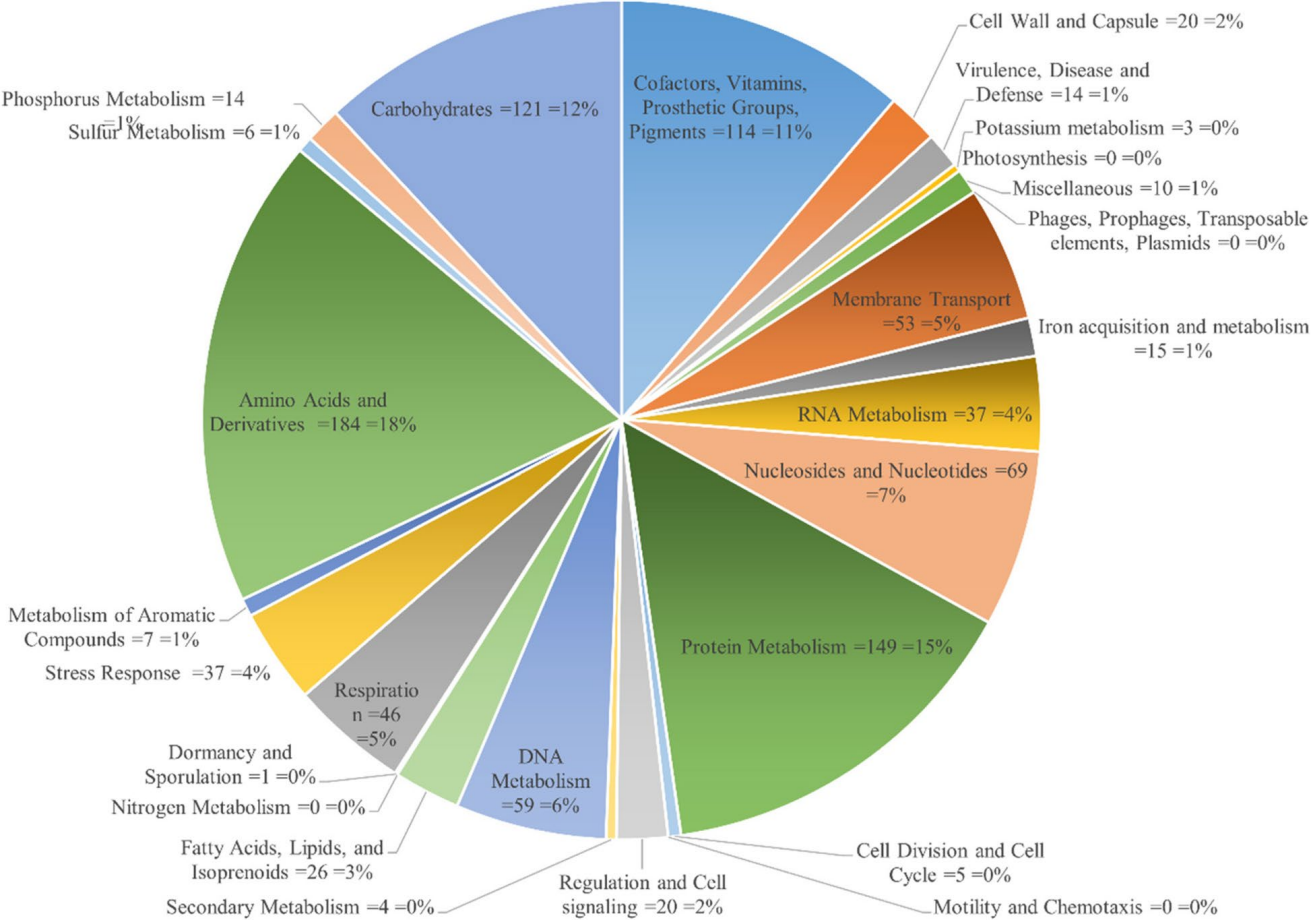
Subsystem features of the core-genome of studied genomes (PM1, PM2, PM3, PVAcc, V1, TX1, Islm, Karachi, BUKK, Faisal, ATTK, Pesh, and Pm70) based on the RAST annotation server

The functional annotation of the core genome in the studied strains reveals that the largest category of *P. multocida* strains comprises 184 genes (18%), which are involved in the biosynthesis of amino acids and their derivatives. Following closely, the second largest category of the core genome consists of 149 genes (15%) associated with protein metabolism, while the category containing genes found in phages, prophages, transposable elements, and plasmids, known as the mobilome, comprises no genes (0%) and stands as the least represented category (Fig. 5). Further analysis revealed that among the core genome of *P. multocida*, a total of 14 genes (1%) were identified to play crucial roles in virulence, disease, and defense mechanisms. Specifically, two genes (*parC*; GO:0003677 and *gyrA*; GO:0003918) were found to confer resistance to fluoroquinolones, while seven genes (*CopZ*; GO:0030001, *CIA*; GO:0004008, *CRD*; GO:0005507, *CcmE*; GO:0017004, *CcmF*; GO:0006461, *ScsB*; GO:0016020, and *ScsC*; GO:0015035) were associated with copper homeostasis and tolerance. Additionally, five genes (*Rv0667*; GO:0003899, *Rv0668*; GO:0003899, *Rv1641*; GO:0003743, *Rv1642*; GO:0003735, and *Rv1643*; GO:0003723) were found to be involved in invasion and intracellular resistance.

The pan-genome analysis identified 23 strain-specific genes (uniquely present) in PM1; however, no unique genes were found in strains PM2 and PM3. Among these genes, one gene (*MO*; GO:0016491) was associated with resistance to a toxic compound, another (*XO*; GO:0004855) with purine utilization, a third (*Val-mt*; GO:0004832) with protein biosynthesis, and finally, one gene (*Ppx*; GO:4309) with phosphorus metabolism. The remaining genes unique to strain PM1 were found to encode hypothetical proteins.

### Presence of genes associated with antimicrobial resistance and virulence

**Fig. 6:**
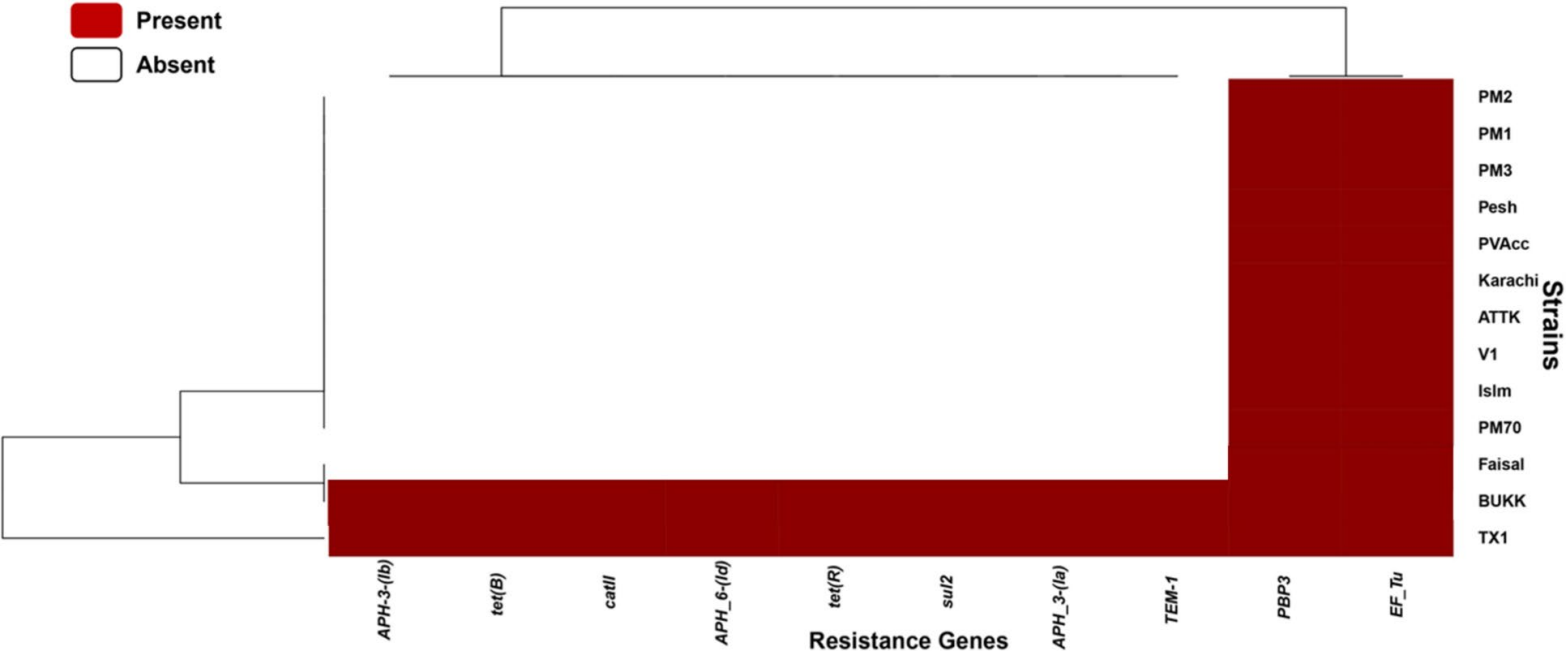
Comparison of resistance genes in Pakistani *P. multocida* and reference strain Pm70

The binary heatmap of the presence and absence of antibiotic resistance determinants shows that a *PBP3* gene conferring resistance to beta-lactam antibiotics and an elfamycin resistance gene *EF-Tu* are present in all the studied genomes (Fig. 6). Whereas, BUKK and TX1 strains harbor additional antibiotic resistance determinants including aminoglycoside resistance genes *aph(3')-la*, *aph(3’’)-Ib,* and *aph(6)-ld*, a *TEM-1* gene conferring resistance to penicillins and the first generation cephalosporins, a plasmid associated gene *sul2* conferring resistance to sulfonamide, a tetracycline resistance gene *tetR*, a chloramphenicol resistance gene *catII*, and a *tet(B)* gene conferring resistance to tetracycline and doxycycline (Fig. 6).

**Fig. 7:**
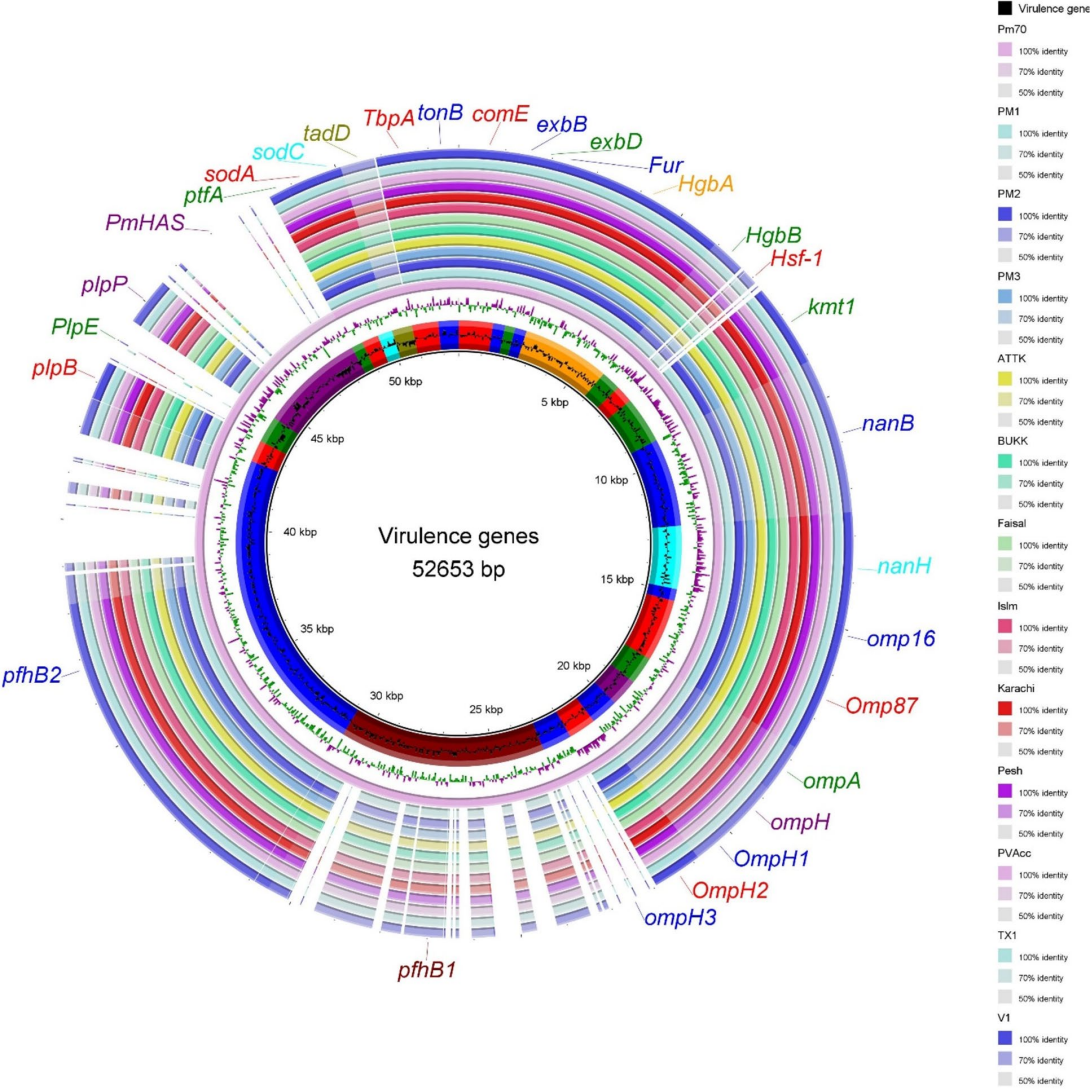
Comparison of virulence genes in Pakistani *P. multocida* strains and the reference strain Pm70. The genomes are presented with different colors in the ring, and the image was generated with BRIG (http://brig.sourceforge.net)

The virulence genes comparison analysis, however, predicted many common genes in Pakistani *P. multocida* and reference strain Pm70 (Fig. 7). These include *HgbA*, *HgbB*, *hsf-1*, *Omp16*, *Omp87*, *OmpH2*, *TbpA*, *PtfA*, *ComE*, *PlpB*, *PlpP*, *PlpD*, *PfhB1*, *PfhB2*, *sodA*, *kmt1*, *tonB*, *nanB*, *nanH*, *exbD*, *fur*, *sodC*, and *exbB* (Table 4). The reference strain Pm70 has additional virulence genes *OmpA, OmpH*, *OmpH1*, *OmpH3*, *PlpE*, *tad*D, and *PmHAS*, which were found absent in all the Pakistani strains (Fig. 7),

## Discussion

HS is an acute, highly lethal, and widespread disease in tropical areas of the world that affects livestock, particularly cattle and buffalo [31]. *P. multocida* serotypes B:2 and E:2 are believed to be the main causes of HS, which develops in the later stages of pasteurellosis. Serotype B:2 is more prevalent in Asian countries including Pakistan than serotype E:2, which is more common in African countries [10]. HS is considered an endemic in Pakistan, where it causes livestock farmers to suffer significant financial losses every year [13, 20]. Hence in this study, we attempted to characterize HS-associated *P. multocida* strains (PM1, PM2, and PM3) from Pakistan at the genomic level. Together with nine other *P. multocida* strains of Pakistani origin and a reference strain Pm70, comparative genomic analysis was performed to better understand the genomic dynamics that may help in genomic epidemiology and reverse vaccinological applications.

The genome size of the sequenced *P. multocida* strains ranges from 2.3 to 2.4 Mbp with G + C contents ranging from 40.3% to 41% which is consistent with previous studies [32]. The MLST genotyping of *P. multocida* is used to determine disease conditions in different hosts such as HS in cattle/buffalo, fowl cholera in poultry, septicaemia in sheep and goats, atrophic rhinitis in pigs, snuffles in rabbits [33]. The MLST scheme assigned ST-44 to all sequenced strains under study, while the in silico RIRDC MLST revealed that all the sequenced strains (PM1, PM2, and PM3) belong to ST-122 and molecular analysis showed that they are serotype B:2. The *P. multocida* isolates with RIRDC ST-122 and multi host MLST ST-44 are strongly associated with bovine HS in Southeast Asia. Notably, *P. multocida* ST-122 serotype B:2 is predominant in Pakistan and has been extensively reported in bovine HS, predominant pasteurellosis in bovine [26, 33].

Bacterial populations frequently exchange genetic determinants such as adhesins, toxins, and antibiotic resistance genes through horizontal gene transfer (HGT). These genetic determinants are found on mobile genetic elements such as plasmids and prophages. Although not all *P. multocida* strains harbor plasmids, plasmids of different sizes (1 up to 100 kb) have been reported in *P. multocida* isolates from various sources [34, 35]. The genome of *P. multocida* PM1, PM2 and PM3 strains sequenced in this study contains no plasmids; however, each strain contains at least two complete prophages. The prophages are hotspots for HGT and the integration of genes associated with stress tolerance, virulence, and antimicrobial resistance [36].

The pan-genome of 13 strains accounted for a total of 2,488 genes, of which 2,187 are core, 93 accessory, and 208 unique genes. The number of pan genes increases with the increase of new genomes, which suggests that the pan-genome of isolates used in the study, is still open. In contrast, the core genes become steady after the addition of certain genomes, which indicates that the core genome is stable. The higher number of core genes and the low number of unique genes in Pakistani strains is indicative of high-level similarity among the strains. This is also supported by phylogenetic analysis of the studied strains i.e., the whole-genome SNPs-based phylogenetic analysis revealed that strains PM1, PM2 and PM3 cluster together nearest to the previously sequenced strain Faisal **(**Fig. 4). Interestingly, the genomes of all Pakistan-origin *P. multocida* strains neatly formed two clades, and the placement of newly sequenced strains in a clade with Faisal strain showed that the Faisal strain might be the ancestor of the newly sequenced strains (PM1, PM2, and PM3) (Fig. 4). The analysis was performed based on whole-genome SNP phylogeny as it gives a better resolution of very closely related organisms entailing more data as compared to core genome phylogeny **(**Fig. 4 and Supplementary Figure S2). It is interesting to mention that PM1 and PM3 have been isolated from buffaloes of the neighbouring cities, similar to that of the Faisal strain (Fig. 4; Table 3). This has been previously reported as well that HS strains formed clades based on their region of isolation (26). However, this might not always be the case as our data has shown HS strains from different regions with different climates might cluster together as well. For example, strains V1, Pesh, Karachi, Islm and ATTK cluster together but they have been isolated from distant cities of different regions **(**Fig. 4**)**. However, further studies on a large set of Pakistani-origin *P. multocida* genomes would be required to confirm this observation. It was further identified that the major content of the core genome is involved in the biosynthesis of amino acids and protein metabolism and few genes of the core genome serve as a part of virulence and defense. Functional annotation of strain-specific genes showed that genes unique to strain PM1 are involved in resistance to copper (*MO*; GO:0016491), purine utilization (*XO*; GO:0004855), protein biosynthesis (*Val-mt*; GO:0004832), and phosphorus metabolism (*Val-mt*; GO:0004832).

**Table 3:** *P. multocida* genomes used in this study

Strain name	Origin	Host	Accession number	Capsular type	Genome size (Mb)	References
PM1	Faisalabad, Pak	Buffalo	JAEMBU000000000	B:2	2.41	This study
PM2	Peshawar, Pak	Buffalo	JAFFJB010000000	B:2	2.3	This study
PM3	Okara, Pak	Buffalo	SDAS00000000	B:2	2.35	This study
PVAcc	Peshawar, Pak	Vaccine strain	JQAD00000000	B:2	2.35	[26]
V1	Lahore, Pak	Buffalo	JQAI00000000	B:2	2.35	[26]
TX1	Rawalpindi, Pak	Buffalo	JQAH00000000	B:2	2.41	[26]
Islm	Islamabad, Pak	Buffalo	JQAB00000000	B:2	2.35	[26]
Karachi	Karachi, Pak	Buffalo	JPHI00000000	B:2	2.37	[26]
BUKK	Bhakkar, Pak	Cattle	JQAO00000000	B:2	2.36	[26]
Faisal	Faisalabad, Pak	Buffalo	JQEB00000000	B:2	2.36	[26]
ATTK	Attock, Pak	Cattle	JQEA00000000	B:2	2.35	[26]
Pesh	Peshawar, Pak	Buffalo	JQAC00000000	B:2	2.35	[26]
Pm70	USA	Chicken	NC_002663	A:3	2.26	[37]

The presence of resistance genes in bacteria suggests their high adaptability in a harsh environment. The Comprehensive Antibiotic Resistance Database (CARD) detected a *PBP3* gene (conferring resistance to beta-lactam antibiotics) and *EF-Tu* (conferring resistance to elfamycin) in all the studied strains including reference strain Pm70. In addition to these two common resistance genes, BUKK and TX1 strains contain several other resistance determinants including aminoglycoside resistance genes *aph(3')-la*, *aph(3’’)-Ib,* and *aph(6)-ld*, a *TEM-1* gene conferring resistance to penicillin and the first-generation cephalosporins, a plasmid associated gene *sul2* conferring resistance to sulfonamide, a tetracycline resistance gene *tetR*, a chloramphenicol resistance gene *catII*, and a *tet(B)* gene conferring resistance to tetracycline and doxycycline. This indicates that BUKK and TX1 strains harbor more resistance-associated genes and are possibly phenotypically resistant to more antibiotics compared to other Pakistani strains and the reference strain Pm70.

It is well known that the expression of various virulent factors of *P. multocida* plays a critical role in pathogenesis. They serve a variety of important roles, including but not limited to adhesion and colonization in the host, enzymatic activity, and iron acquisition, etc*.* that leads to the development of pasteurellosis and persistence in the host environment [38]. The most important VFs include capsules, lipopolysaccharides (LPS), outer-membrane proteins (OMPs), and fimbriae. Furthermore, some VFs are believed to be a major contributing element to HS pathogenesis and all serotype B:2 strains contain *ptfA* encoding type 4 fimbriae, *OmpH and Oma87* encoding outer membrane proteins, *pfhB* encoding filamentous hemagglutinin, *tbpA* encoding a transferrin-binding protein, *sodC* encoding superoxide dismutase, *hgbA* encoding a haemoglobin binding protein, and *nanH* encoding neuraminidases [10, 39].

Bacterial adhesion and colonization to the epithelial surface are essential to establish infection [3]. In our study VFs such as *ptfA* (encoding type 4 fimbrial subunit), *PfhB1*, *PfhB2* and *hsf-1* were present in all the studied genomes. They are important in surface adhesion and play a major role in the colonization of the upper respiratory tract. Iron is essential for bacterial growth and survival in the host. *P. multocida* has evolved multiple mechanisms for iron uptake. We found many genes (*tbpA*, *HgbA*, *HgbB, fur, exbD, exbB,* and *tonB*) in our studied genomes encoding proteins with predicted roles in iron acquisition and iron transport [4]. VFs encoding extracellular enzymes such as neuraminidase (*nanB* and *nanH*) were also found in the studied strains. These VFs are reported to enhance metabolic activity, and are critical for the pathogenesis of *P. multocida* in the host. The superoxide dismutase genes (*sodA* and *sodC*), which play an important role in protection against oxidative stress are also found present in all the analyzed strains. Outer membrane proteins are the main component of the bacterial outer membrane and are critical for infection and pathogenesis. Notably, *Omp16*, *Omp87*, *OmpH2, PlpB*, *PlpP*, and *PlpD* were uniformly present across all the studied genomes. These results are consistent with earlier studies [40, 41].

Surprisingly, *OmpH*, *OmpH1*, *OmpH3*, and *PlpE* were not found in any Pakistani *P. multocida* strains while these surface antigens are often used in vaccine studies. For instance, OmpH [42] and PlpE [43, 44] conferred protective immunity against *P*. *multocida* infection. A few previous studies reported the presence of the *plpE* gene across different strains of *P. multocida*, irrespective of serotypes [45, 46]. For example, there was high *plpE* gene sequence homology (˃ 90%) among Indian isolates (A: 3. B: 2 and D: 1) and the reported sequences from other serotypes [X-73 (A: 1), P-470 (A: 3), P-1059 (A: 3)] of *P. multocida* indicating the universal presence of *plpE* gene across all serotypes [45]. However, recent comparative genomic studies, including our present investigation, contradict the previous findings and suggest that outer membrane protein PlpE was not identified in any genome from capsular serogroup B. Instead, it is significantly associated with capsular serogroup A and F [29]. Similarly, outer membrane proteins OmpH1 and OmpH3 are significantly associated with capsular serogroup A as compared to all other serogroups [5, 29].

Beyond the mere presence of particular VFs in the genomes, their expression levels play an important role in determining pathogenicity and disease manifestation in the host. When conducting a comparative genome analysis between the highly virulent strain PmCQ2 and naturally attenuated strain PmCQ6 of *P. multocida* (serotype A), it was reported that they exhibited high genome similarity (99%), sharing common virulent factors. The differences in pathogenicity between the two strains was attributed to the differential expression of virulence genes [47]. Similarly, molecular variations within virulence-associated genes may affect both host specificity and virulence. Structural characterization of an abundant outer membrane protein A (OmpA) in *P. multocida* revealed the presence of four hypervariable extracellular loops, predicted to be more antigenic in bovine isolates compared to porcine isolates [48]. These loop regions, which contain charged residues, are suggested to be important for adherence to the host cell and potentially play an important role in the development of HS in bovine. Hence, many factors, including molecular-level variation within VFs, their presence or absence, and most importantly their differential expression, collectively contribute to shaping the pathogenicity of bacterial strains.

## Conclusions

This study comprised a comparative genomic analysis of three novel strains of HS-associated *P. multocida* serotype B:2 with nine publicly available Pakistani origin *P. multocida* strains and a reference strain Pm70. The core-genome SNPs-based phylogenetic analysis indicated a close resemblance among Pakistani strains and few genes (1%) of the core-genome serve as a part of virulence and defense. Surprisingly, VFs like *OmpH* and *PlpE* were not found in any Pakistani *P. multocida* strains while these surface proteins are used in vaccine studies and reported to provide protective immunity. Therefore, this study warrants further research to investigate the diversity and prevalence of *PlpE* and *OmpH* genes in HS-associated Pakistani *P. multocida* strains to develop domestic HS-associated *P. multocida* specific therapeutics.

## Methods

### Bacterial strain isolation and characterization

*Pasteurella multocida* isolate PM1 was isolated from the heart blood of an infected buffalo in Faisalabad, PM2 from the blood of an infected buffalo in Peshawar, and PM3 was isolated from a buffalo (PM3) died apparently with the symptoms of HS in Okara, Pakistan. The morphological characteristics of the isolates were observed by growing on BHI and MacConkey agar by incubating at 37 ℃ for 16–18 h. The genomic DNAs (gDNAs) were extracted from purified cultures using GeneJET Genomic DNA Purification Kit, cat # K0721 (Thermo Fisher). The extracted gDNAs were quantified using nanodrop (NanoDrop 2000c, Thermo Scientific), and the integrity and purity of the extracted gDNAs were tested through agarose gel electrophoresis. The serotypes were confirmed by molecular analysis of gDNAs through species-specific and type-specific PCR [49]. The *kmt*1 gene was amplified using KMT1SP6-KMT1T7 primers that identify *P. multocida* species (species-specific) whereas the 6B gene was amplified using KTSP61-KTT72 primers that distinguish HS-associated *P. multocida* serotype B:2 strains (type-specific).

### Genome sequencing and annotation

The whole-genome shotgun sequencing of newly isolated *P. multocida* strains PM1, PM2 and PM3 was achieved using Illumina HiSeq 2500 platform. The sequence reads were trimmed using Trimmomatic 0.30 [50], and the trimmed reads were de novo assembled using SPAdes (version 3.12.0) [51]. The generated contigs were annotated by Prokka at default parameters [52].

### MLST genotyping and identification of prophage regions and plasmids

The in silico MLST genotyping was performed at PubMLST using the *P. multocida* typing database (https://pubmlst.org/bigsdb?db=pubmlst_pmultocida_seqdef) [53]. The PubMLST hosts two separate MLST schemes (Multi-host MLST and RIRDC MLST) for *P. multocida*. The multi-host MLST scheme includes isolates from a range of hosts such as birds, pigs, sheep, and cattle, and is based on seven housekeeping genes (*adk*, *aroA*, *deoD*, *gdhA*, *g6pd*, *mdh*, and *pgi*). The RIRDC MLST scheme is based on seven housekeeping genes (*adk*, *est*, *pmi*, *zwf*, *mdh*, *gdh*, and *pgi*) developed to investigate avian isolates.

The prophages in the sequenced genomes were identified and annotated using PHASTER [54] and the plasmid replicons (*rep*) were identified by PlasmidFinder 2.1 at the default parameters [55].

### Pan-genome estimation and phylogenetic relationships

The Pakistani origin *P. multocida* genomes (*n* = 9) available at NCBI were downloaded with a reference strain Pm70. In addition, three genomes of *P. multocida* (sequenced in the current study) were also included (Table 3**)**. To proceed with pan-genome analysis all selected genomes were first annotated by Prokka at default parameters [52]. The pan-genome analysis and core-genome SNPs based phylogenetic relationship were inferred using the in-house pipeline PanRV [56]. For, whole-genome SNPs-based phylogenetic analysis, the genomes were uploaded to the online server CSI Phylogeny 1.4 hosted at https://cge.food.dtu.dk/services/CSIPhylogeny/ [57] with the following default setting: minimum depth at SNP positions 10, relative depth at SNP positions 10, the minimum distance between SNPs (prune) 10, minimum SNP quality 30, and minimum Z-score of 1.96. The SNP calling was performed against *P. multocida* strain PVAcc (serotype B:2) and a maximum likelihood tree was generated using FastTree 2 tool [58].

### Functional annotation of core and unique genes

The functional annotation of core and unique genes was achieved using Rapid Annotation using Subsystem Technology (RAST), available at https://rast.nmpdr.org/rast.cgi [59]. RAST conducts BLAST search against the SEED database and provides high-quality functional annotations [60].

### Comparison of genes associated with antimicrobial resistance and virulence

The acquired genes and chromosomal mutations conferring antibiotic resistance were identified using CARD [61]. For the identification of virulence-associated genes, 29 known *P*. *multocida* VFs were retrieved from the NCBI database (Table 4). The BLASTp search was performed to identify the VFs in the selected genomes based on sequence similarity (genes exhibiting < 80% sequence similarity were deemed present). Subsequently, a comparative analysis of VFs with the reference strain Pm70 was performed using BRIG (Blast Ring Image Generator) [62].

**Table 4:** Selected VFs for comparative analysis of Pakistani *P. multocida* and reference strain Pm70

Protein	Description	Accession number	Number of a.a
HgbA	Iron acquisition factor A	AAQ14873.1	971
HgbB	Iron acquisition factor B	APX53047.1	251
Omp16	Outer membrane protein 16	AHW46109.1	150
Omp87	Outer membrane protein 87	ANJ20895.1	791
OmpA	Outer membrane protein A	AFS89615.1	352
OmpH	Outer membrane protein H	AFU91512.1	348
OmpH1	Major outer membrane protein OmpH-1	SNV59447.1	348
OmpH2	Major outer membrane protein OmpH-2	VEJ48890.1	350
OmpH3	Major outer membrane protein OmpH-3	AMK08231.1	372
TbpA	Iron acquisition related factor	AAK02460.1	334
PtfA	Adhesins (type IV fimbriae)	AKO69808.1	144
ComE	comE protein	AMK08712.1	434
PlpE	Protective outer membrane lipoprotein E	AAK03601.1	335
PlpB	Protective outer membrane lipoprotein B	AAK03814.1	276
PlpP	Protective outer membrane lipoprotein P	AAK03602.1	348
PfhB1	Adhesins	AAK02141.1	2615
PfhB2	Adhesins	AAK02143.1	3919
Soda	Superoxide dismutases	AMK08395.1	214
SodC	Superoxide dismutases	AAK04036.1	186
kmt1	*Pasteurella multocida* specific	AAK03445.1	599
tonB	Iron acquisition related factor	ART94483.1	256
nanB	Sialidase NanB	AAG35309.1	1070
nanH	Sialidase NanH	SNV63299.1	798
exbD	Iron acquisition related factor	AAR87755.1	129
Fur	Iron acquisition related factor	AAK02436.1	146
exbB	Iron acquisition related factor	AAQ05037.1	152
hsf-1	Adhesins	AQM74573.1	217
tadD	putative nonspecific tight adherence protein D	AAF68419	257
PmHAS	hyaluronan synthase	AAC38318	972

### Supplementary Information

**Additional file 1:** **Figure S1.** Molecular characterization of P. multocida isolates (PM1, PM2 and PM3). (A) The amplified product of kmt1 gene using Pasteurella species-specific primers. (B) amplified product of 6B gene using HS causing P. multocida B:2 type-specific primers. **Figure S2.** Core-genome SNPs-based phylogenetic relationship of the sequenced strains (PM1, PM2, and PM3) with 9 other Pakistani P. multocida strains (PVAcc, V1, TX1, Islm, Karachi, BUKK, Faisal, ATTK, and Pesh) and a reference strain Pm70.

## Data Availability

The whole-genome sequence data of PM1, PM2, and PM3 have been deposited at DDBJ/ENA/GenBank under the accession number JAEMBU000000000, JAFFJB000000000, and SDAS00000000, respectively.

## Abbreviations

HS: Haemorrhagic septicaemia
BRD: Bovine respiratory disease
FC: Fowl cholera
AR: Atrophic rhinitis
CDS: Coding sequences
MLST: Multi-locus sequence typing
HGT: Horizontal gene transfer
CARD: Comprehensive antibiotic resistance database
COGs: Cluster of orthologous genes
FAM: Functional annotation module
SNP: Single nucleotide polymorphism
ICE: Integrative conjugative element
VFs: Virulence factors
LPS: Lipopolysaccharides
OMPs: Outer-membrane proteins
BHI: Brain heart infusion
gDNA: Genomic DNA
