# Supplementary material for: Genomic characterization and comparative genomic analysis of HS-associated Pasteurella multocida serotype B:2 strains from Pakistan
Source: BMC Genomics. 2023 Sep 14;24:546. doi: 10.1186/s12864-023-09626-5 (PMC10500850; doi:10.1186/s12864-023-09626-5)
Supplement: Supplementary file 1 — Additional file 1: Figure S1. Molecular characterization of P. multocida isolates (PM1, PM2 and PM3). (A) The amplified product of kmt1 gene using Pasteurella species-specific primers. (B) amplified product of 6B gene using HS causing P. multocida B:2 type-specific primers. Figure S2. Core-genome SNPs-based phylogenetic relationship of the sequenced strains (PM1, PM2, and PM3) with 9 other Pakistani P. multocida strains (PVAcc, V1, TX1, Islm, Karachi, BUKK, Faisal, ATTK, and Pesh) and a reference strain Pm70. [file 12864_2023_9626_MOESM1_ESM.pdf]

Supplementary Information

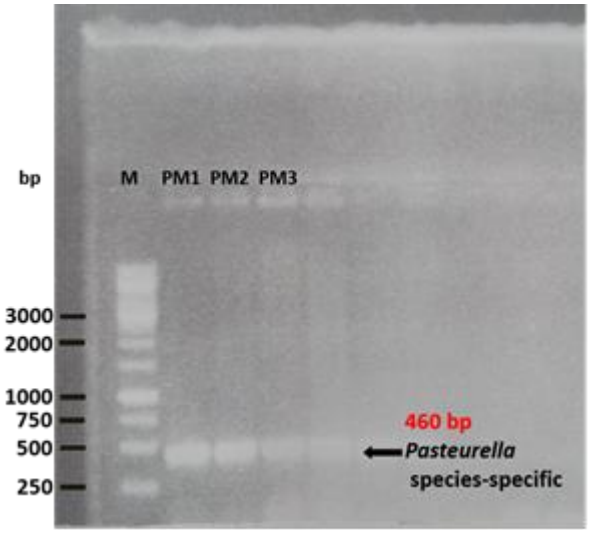

Supplementary Figure S1: Molecular characterization of *P. multocida* isolates (PM1, PM2 and PM3). (A) The amplified product of *kmt*1 gene using *Pasteurella* species-specific primers

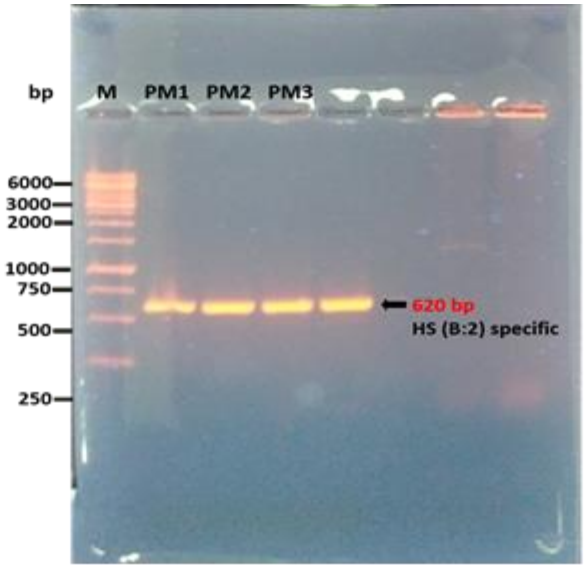

Supplementary Figure S1: Molecular characterization of *P. multocida* isolates (PM1, PM2 and PM3). (B) amplified product of 6B gene using HS causing *P. multocida* B:2 type-specific primers.

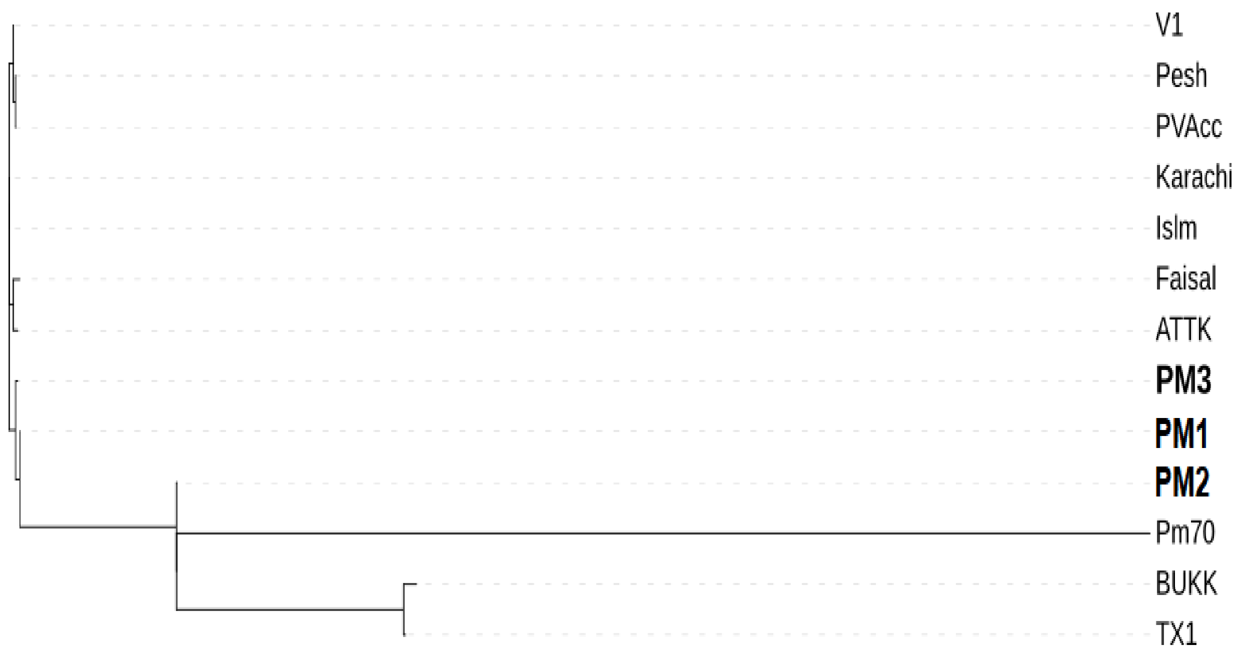

Supplementary Figure S2. Core-genome SNPs-based phylogenetic relationship of the sequenced strains (PM1, PM2, and PM3) with 9 other Pakistani *P. multocida* strains (PVAcc, V1, TX1, Islm, Karachi, BUKK, Faisal, ATTK, and Pesh) and a reference strain Pm70.
